# MALDI-TOF MS in conjunction with machine learning: toward a new era for antimicrobial susceptibility testing

**DOI:** 10.3389/fcimb.2025.1731083

**Published:** 2026-01-23

**Authors:** Miao Wang, Wei Xia, Jia Du, Hanshuang Ma, Baoyu Sun, Huabin Jiang, Jiancheng Xu

**Affiliations:** 1 Department of Laboratory Medicine, Center of Infectious Diseases and Pathogen Biology, The First Hospital of Jilin University, Changchun, Jilin, China; 2 College of Medical Technology, Beihua University, Jilin, Jilin, China

**Keywords:** drug resistance, machine learning, MALDI-TOF MS, microbial sensitivity tests, microbiological techniques

## Abstract

Global public health is formidably threatened by antimicrobial resistance (AMR). Antimicrobial susceptibility testing (AST) is characterized by its long duration. Matrix-assisted laser desorption/ionization time-of-flight mass spectrometry (MALDI-TOF MS) is notable for its rapid analysis and cost-effectiveness. However, its role in AST has not been fully explored. In recent years, new opportunities for predicting AMR using MALDI-TOF MS data have been provided by the development of machine learning (ML) technologies. The research progress in using MALDI-TOF MS combined with ML for AMR testing is surveyed by this review, and critical steps including raw MALDI-TOF MS data acquisition, raw data preprocessing, algorithm selection, hyperparameter optimization, among others. It was found by us that the true resistance status can be comprehensively reflected by large-scale datasets, but effective management of high-dimensional data challenges is required. Algorithm performance can be enhanced by identifying the optimal combination of hyperparameters. Better predictive performance than individual models can be achieved by stacking ensemble learning methods. Model performance and generalizability can be more effectively assessed by metrics such as the Area Under the Receiver Operating Characteristic Curve (AUROC). The decision-making process can be understood by users with the help of model interpretation, thereby increasing model transparency and acceptability. Insufficient sample size, inadequate data standardization, and limited model generalizability are included in the current challenges. Continuously optimized, the integration of MALDI-TOF MS and ML is poised to open future avenues for rapid and accurate AMR prediction.

## 1 Introduction

A formidable global public health security challenge has been posed by Antimicrobial Resistance (AMR). The impact of AMR can be mitigated by the use of effective and timely surveillance, which is a pivotal strategy (Ho et al., 2025). Bacterial growth, a process that typically requires 24–48 hours, is relied upon by conventional antimicrobial susceptibility testing (AST), thereby often causing a delay in the initiation of optimal antimicrobial therapy. Resistance genes can be rapidly detected directly from samples by gene-based AST methods, but these methods are often confined to single-target genes, a narrow focus on resistance mechanisms is exhibited, and high costs are entailed (Weis et al., 2022). A turnaround time of approximately 38 minutes per analysis is achieved by Matrix-assisted laser desorption/ionization time-of-flight mass spectrometry (MALDI-TOF MS), with a cost of $0.50 per test. It has emerged as a widely adopted method for microbial identification in clinical laboratories (Dhiman et al., 2011). Despite its demonstrated utility in bacterial identification, the application of MALDI-TOF MS in AST remains in its exploratory stage. To maximize the utility of MALDI-TOF MS data and facilitate timely clinical decision-making, machine learning (ML) algorithms have been used by researchers to transform raw data into predictive models for AST (Weis et al., 2020).

A comprehensive literature search on PubMed, Web of Science, and Scopus was conducted by us to investigate the research progress of AST using MALDI-TOF MS data in conjunction with ML algorithms and to analyze the application potential of this method. Keywords such as “Drug Resistance” “Antimicrobial Stewardship” “Microbiological Techniques” “Microbial Sensitivity Test” “MALDI-TOF MS” “Artificial Intelligence” and “Machine Learning” were employed by us. References were selected based on their relevance and significance to the topic of this review.

## 2 MALDI-TOF MS-based AST methods

A highly efficient and sensitive analytical technique for the identification and characterization of microorganisms is served by MALDI-TOF MS. The rapid and precise determination of microbial species is facilitated by this method through the analysis of bacterial extracts and the generation of unique microbial mass spectral fingerprint profiles (Chen et al., 2023). Nevertheless, limitations are still present with MALDI-TOF MS in AST (Oviaño and Bou, 2019). Several MALDI-TOF MS-based AST methods have been previously proposed. For example, bacterial resistance is evaluated by enzyme activity assays through monitoring the decrease in peaks corresponding to antimicrobial agents and the appearance of peaks for hydrolysis products; Bacterial growth is detected and antimicrobial susceptibility is determined by the Direct On-chip Targeted Microdroplet Growth Assay (DOT-MGA) through the comparison of mass spectrometry peaks obtained from bacterial cultures incubated in the presence and absence of antimicrobial agents; Bacterial metabolic activity is monitored through the incorporation of stable isotope-labeled substrates into the culture medium, which facilitates the detection of AMR by the isotopic labeling method; Bacterial resistance is quantitatively and qualitatively determined by the Minimum Profile Change Concentration (MPCC) method through the identification of the lowest antimicrobial concentration that elicits significant changes in the mass spectrometry profile, as compared across different antimicrobial concentrations; Bacterial resistance is evaluated by the MALDI Biotyper Antibiotic Susceptibility Testing Rapid Analysis (MBT-ASTRA) method through comparing the AUC of mass spectra from bacteria exposed and unexposed to antimicrobial agents, thereby providing insights into metabolic activity and protein expression under specific conditions (Florio et al., 2020; Idelevich and Becker, 2021). Although certain advantages in AST are offered by these methods, their application is restricted by inadequate standardization, subjective interpretation of results, and limited ability to detect complex resistance mechanisms.

## 3 Machine learning modeling workflow based on MALDI-TOF MS data

**Figure 1:**
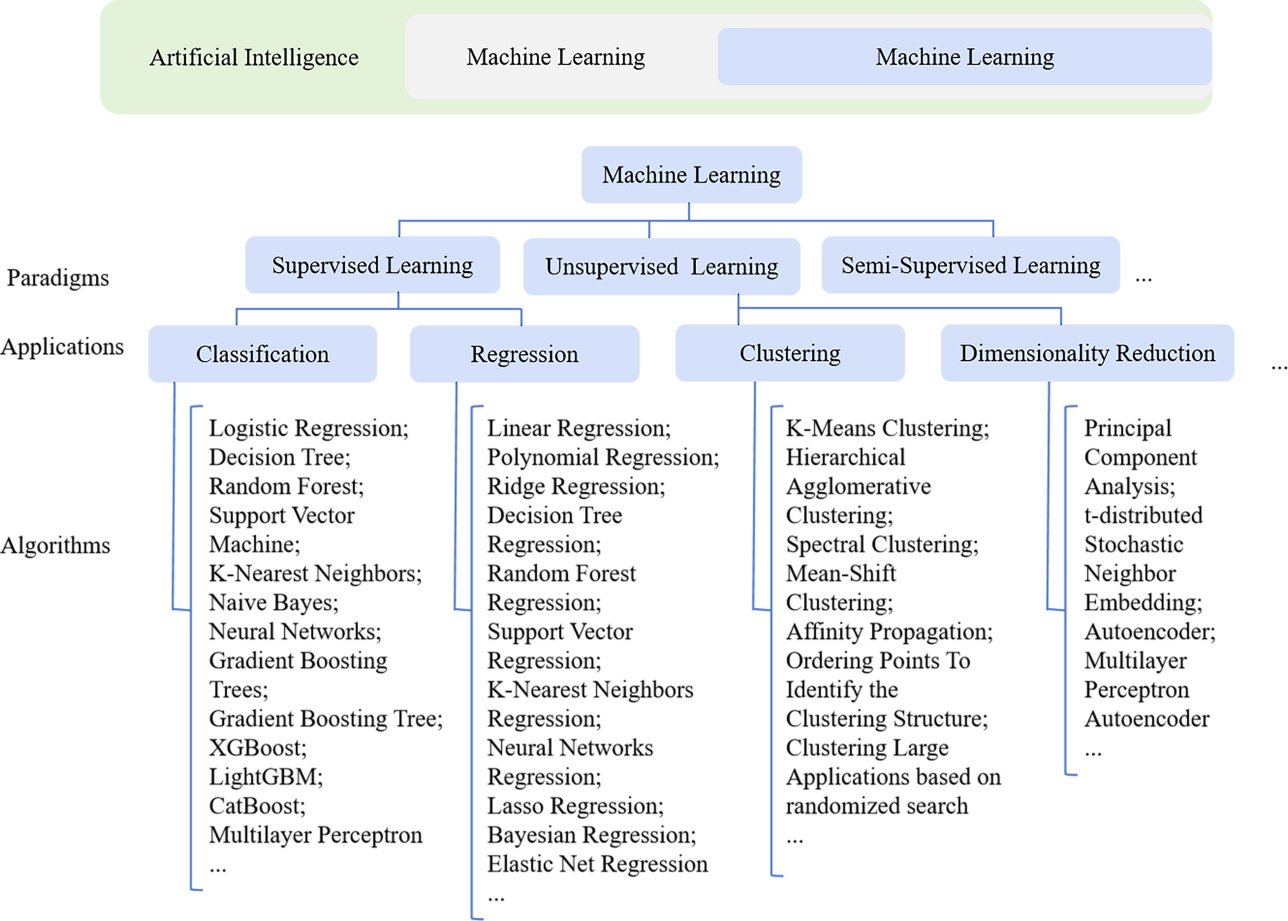
The relationship among artificial intelligence, machine learning, and deep learning, and the categorization of machine learning paradigms and associated algorithms.

**Figure 2:**
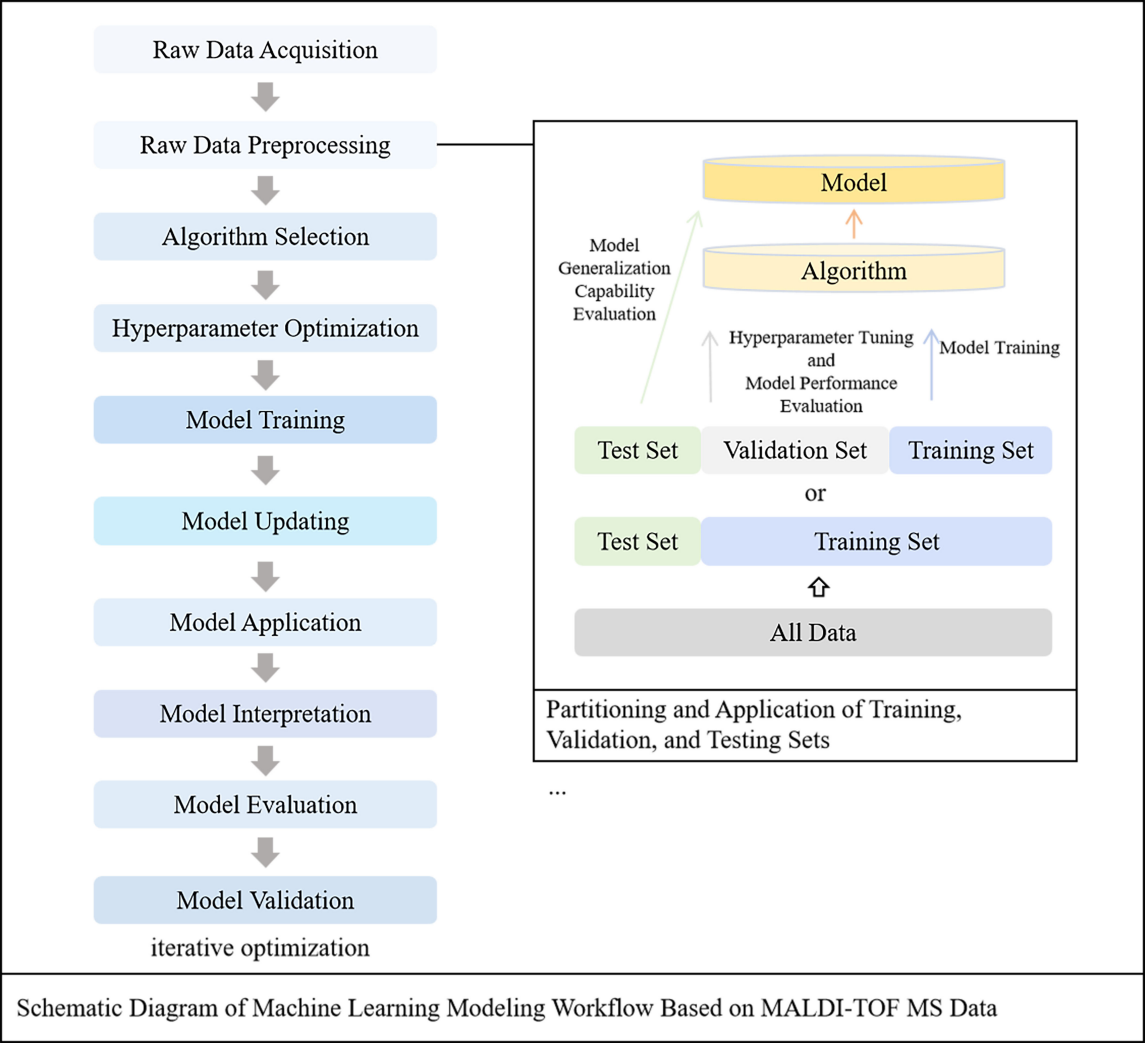
Schematic workflow of MALDI-TOF MS machine-learning modeling and the partitioning and use of training, validation and test sets.

As the digitalization and availability of information increase, Artificial Intelligence (AI) has become a prominent topic in the field of medicine (Deo, 2015). The objective of AI is that computer systems are enabled to perform tasks that typically require human intelligence. This objective is achieved through the employment of specific methodologies such as ML and Deep Learning (DL), which are considered subdomains within the broader field of AI (Choi et al., 2020) **Figure 1**. ML is situated at the intersection of statistics, where relationships within data are aimed to be uncovered, and computer science, where efficient algorithms are focused on being developed (Tur, 2021). By learning patterns and regularities from data, computers are enabled by ML to make predictions or decisions, making it highly suitable for analyzing complex datasets (Goodswen et al., 2021). Compared with ML, DL is distinguished by a greater number of node layers and a larger overall network scale, which enables it to more accurately represent complex interrelationships (Handelman et al., 2018). The capability to predict AMR has been demonstrated by ML models derived from MALDI-TOF MS data, which is considered a promising approach for rapid and accurate diagnostics **Figure 2**.

### 3.1 Raw data acquisition

The generalizability of the model is often diminished as a result of the limited sample data size (Weis et al., 2020). The true prevalence of drug resistance can be more comprehensively captured by large-scale datasets. Consequently, extensive datasets are often necessitated by state-of-the-art DL architectures to achieve optimal performance. However, particular attention is warranted by the phenomenon of high-dimensional datasets (Nguyen et al., 2024). It is noteworthy that it has been demonstrated by Weis et al. that a significant impact on the performance of predictive models is exerted by the temporal origin of the samples, specifically the year of collection. The predictive accuracy of the models can be markedly improved by employing spectral data derived from samples collected in more recent years (Weis et al., 2022). High-quality MALDI-TOF MS data needs to be utilized to optimize model training.

Pure bacterial cultures grown on solid media are typically employed in MALDI-TOF MS analysis. The phenotypic characteristics of bacteria under different culture conditions can be significantly influenced by the growth temperature. Degraded mass spectra due to bacterial lysis may be caused by frozen and lyophilized cultures, whereas clearer and more reproducible mass spectral results are generally obtained from fresh cultures (Topić Popović et al., 2023). It was demonstrated by GATO et al. that the expression of peptides and proteins during bacterial growth is significantly impacted by the nutritional composition of different culture media. In their study, the prediction outcomes for carbapenemase - producing *Klebsiella pneumoniae* were notably influenced by this variation (Gato et al., 2023). The accuracy and reproducibility of experimental results are underscored by the essential role of consistent culture conditions. Additionally, a variety of pre-analytical processing methods are available for cultures. In the direct deposition method, colonies are simply spotted onto the target plate and then covered with the matrix. This method is characterized by low labor intensity and rapid operation. If an additional protein extraction step is implemented, it would involve bacterial lysis, followed by centrifugation, washing, and concentration. While a more purified protein profile is achieved by this approach, thereby enhancing data quality, its use in clinical settings is typically precluded by its labor-intensive, time-consuming, and costly nature. Discrepancies in peak detection may be led to by variations in pre-analytical processing methods. In the *Escherichia coli* gentamicin model examined by Maureen Feucherolles et al., a lower model performance, with an Area Under The Precision-Recall Curve (AUPRC) of 0.23, was resulted in by the ethanol/acetonitrile extraction method (EtOH/ACN). In contrast, a higher performance, with an AUPRC of 0.92, was achieved by the direct deposition method (Feucherolles et al., 2021). Consequently, optimal results are more likely to be achieved when mass spectra are acquired under standardized culture conditions and sample preparation protocols. Drug resistance was not directly assessed by using antimicrobial agents by Margot Delavy et al., but was rather indirectly evaluated by inducing differential protein expression. In *Candida albicans*, the calcineurin signaling pathway, which is responsible for mediating the fungal stress response and regulating drug resistance, may have distinct activities exhibited between fluconazole-resistant and susceptible strains. The normal function of this pathway can be disrupted by cyclosporin A, a calcineurin inhibitor. Consequently, distinct protein expression profiles between resistant and susceptible strains can emerge following treatment with cyclosporin A (LaFayette et al., 2010). Without directly employing fluconazole, differences in protein expression between fluconazole-resistant and susceptible strains following treatment with cyclosporin A were detected by Margot Delavy et al. using MALDI-TOF MS. By integrating ML algorithms for rapid classification, rapid (4h) and accurate prediction of fluconazole resistance was successfully achieved (Delavy et al., 2019).

Owing to their limited resolution, proteins or molecules that fall outside the conventional mass - to - charge ratio (m/z) range of 2000–20000 cannot be accurately identified by entry - level MALDI - TOF MS instruments. Certain AMR - associated proteins with low expression levels may be obscured by background noise in the conventional spectra, thereby impeding their effective detection (Yoon and Jeong, 2021).

### 3.2 Raw data preprocessing

The intricacy of algorithmic processing can be exacerbated, the precision of predictive modeling can be diminished, and the duration required for model training can be significantly extended by the considerable noise and extraneous information contained within raw datasets. Before ML techniques are employed for the analysis of MALDI-TOF MS data, data preprocessing must be conducted to enhance data quality and analyzability. Intensity variance stabilization, smoothing, baseline removal, total ion current normalization, mass spectral trimming, and the association with corresponding resistance labels are included in the preprocessing steps. The quality of peaks (features) utilized in ML models is influenced by the distinct preprocessing methods employed (Chung et al., 2023a). Moreover, the selection of preprocessing methods is determined by the underlying data structure and the architecture of the anticipated model.

#### 3.2.1 Handling class imbalance

The substantial disparity in the number of instances among different classes within a dataset is referred to as class imbalance, which is a prevalent issue in ML classification tasks. The predictive accuracy for the minority class is often diminished as models tend to exhibit a bias towards the majority class due to this imbalance (Andaur Navarro et al., 2023). Class imbalance is addressed by resampling techniques, which represent one of the primary approaches. At the data level, techniques such as oversampling the minority class and undersampling the majority class are included. The number of samples across different classes in a dataset is balanced by the Synthetic Minority Over-sampling Technique (SMOTE) through synthesizing new minority class samples. However, in the study by López-Cortés XA et al., it was found that SMOTE did not improve performance but instead generally led to degraded model performance (López-Cortés et al., 2025). This may be attributed to the fact that MALDI-TOF MS data with high-dimensional sparsity is not well-suited for SMOTE. The distribution characteristics of MALDI-TOF MS data may not be conformed to by the synthetic samples generated by SMOTE, or noise may be introduced. Moreover, substantial information loss may result from undersampling (Ren et al., 2024).

#### 3.2.2 Dataset binning

To facilitate data processing for ML, MALDI-TOF MS data was transformed into fixed-length vectors by López-Cortés XA et al. Continuous m/z values were binned, and the mean intensity within each bin was calculated as the representative value. This method retains sufficient information while reducing the computational burden on the model (López-Cortés et al., 2025). A dynamic binning feature engineering technique that adaptively adjusts bin width according to the data distribution was developed by Nguyen H-A et al. Narrow bins were utilized in regions with high peak density to capture fine-grained information, while wider bins were applied in sparse regions to minimize redundant features. Feature dimensionality was effectively reduced, the flexibility and efficiency of feature extraction were enhanced, and thereby the handling of MALDI-TOF MS data was improved by this approach (Nguyen et al., 2024). The efficacy of dynamic binning across diverse ML models could be further explored in future investigations.

#### 3.2.3 Feature selection

Not every mass-to-charge (m/z) ratio value generated by MALDI-TOF MS is corresponded to by proteins associated with bacterial antibiotic resistance. Extended algorithm training times and diminished model prediction accuracy can be caused by the presence of an excessive number of irrelevant features in the dataset. Therefore, the most informative features need to be selected for the algorithm, and those identified as less important need to be iteratively removed. The computational time required for model training is not only reduced by this strategy, but overfitting is also effectively prevented by it. As a result, the model’s classification accuracy on unseen data is thereby enhanced. The model using random forest for feature filtering, which was trained by Ren M et al., outperformed other models. SHapley Additive exPlanations (SHAP) analysis can be used for subsequent model interpretation but was not employed for feature selection. The computational time required for SHAP analysis is significantly increased with each additional feature, and it is rendered impractical for extremely high-dimensional datasets. Preliminary feature filtering was sufficiently performed by Random forest, and the number of features was reduced to a range more effectively manageable by SHAP analysis (Ren et al., 2024).

#### 3.2.4 Dataset partitioning

Following raw data preprocessing, each mass spectrometry file is categorized into the corresponding antimicrobial drug sets based on its features. To ensure that the proportion of resistant and susceptible strains in the training, validation, and test sets mirrors that in the original dataset, each antimicrobial drug set can be stratified into training, validation, and test sets according to a predefined ratio. Typically, the training dataset is utilized to develop predictive models for antimicrobial drugs. The hyperparameters are optimized and preliminary performance assessments of these models are conducted by means of the validation dataset. Ultimately, the test dataset is employed to conduct the final evaluation and validation of the models **Figure 2**. When the training dataset is of limited size, the noise in the data may be learned by the model rather than the genuine distribution of the data, which can lead to overfitting (Zhang et al., 2023).

In contrast to relying exclusively on a single characteristic peak, more comprehensive molecular features are afforded by the ensemble of multiple characteristic peaks, which is markedly improved in terms of the model’s comprehensiveness, robustness, and accuracy in classification tasks (Wang et al., 2018b). Simultaneously, the generalization capability of the model can be greatly enhanced by the standardization of spectral acquisition and preprocessing procedures (López-Cortés et al., 2025).

### 3.3 Algorithm selection

Supervised learning, unsupervised learning, semi-supervised learning, and reinforcement learning are typically encompassed by ML algorithms **Figure 1** (Goodswen et al., 2021). A model is trained on a training dataset that includes input features and corresponding target labels by supervised learning. The relationship between these features and labels is learned by the model, which enables it to generalize and predict the output for unseen data. Various learning tasks, including classification and regression, are widely applied in supervised learning (Jiang et al., 2020; Wang et al., 2023). Prior knowledge is not relied upon in the operation of unsupervised learning. The intrinsic structure, patterns, and distributional relationships within the data are autonomously uncovered by the algorithm through processing unlabeled data. This approach is well-suited to learning tasks such as clustering and dimensionality reduction (Bröker et al., 2024). A small amount of labeled data along with a large quantity of unlabeled data is employed by semi-supervised learning. The model’s performance is enhanced by utilizing the structural information within the unlabeled data. In contrast, pre-labeled data is not required by reinforcement learning. Feedback from the environment, in the form of rewards or penalties, is relied upon to guide the learning process. The objective is for an optimal policy for acting within the environment to be learned in order to maximize cumulative rewards (Rani et al., 2023).

The integration of MALDI-TOF MS with ML for AMR prediction is fundamentally based on supervised learning methodologies (Jiang et al., 2020). In the preprocessing stage, such as in feature selection and dimensionality reduction, unsupervised learning is often employed. When training data are limited, simple algorithms with regularization, such as linear regression and logistic regression with penalty terms, are usually chosen. The training set may have limited fitting capacity by these algorithms, resulting in higher error rates (high bias), but better generalization ability (low variance) is typically exhibited. When training data are abundant and complex feature interactions may be involved by the underlying algorithms, instance-based methods (e.g., k-nearest neighbors) or tree-based algorithms (e.g., stochastic gradient boosting or random forests) may be performed well, characterized by low bias and high variance (Deo, 2015).

The intrinsic complex nonlinear relationships inherent in MALDI-TOF MS data have been proven to be effectively addressed by the gradient boosting technique utilized by the CatBoost algorithm. As reported by López-Cortés et al., in the prediction of ciprofloxacin-resistant *Escherichia coli*, an Area Under The Receiver Operating Characteristic Curve (AUROC) and an AUPRC of 0.91 were achieved by the algorithm, with balanced accuracy and F1 score values of 0.81 and 0.78, respectively. Similarly, in the prediction of oxacillin-resistant *Staphylococcus aureus*, an AUROC of 0.86, an AUPRC of 0.73, balanced accuracy of 0.77, and an F1 score of 0.61 were demonstrated by the algorithm (López-Cortés et al., 2025). Models with strong generalizability and adaptability may be trained in the future, thereby enhancing the capability for detecting AMR **Table 1**.

**Table 1:** Model indicators in the relevant literature.

Model	Bacteria	Antibiotics - AUROC metric
Logistic Regression	Staphylococcus aureus	Methicillin-0.979 (Yong et al., 2025); Levofloxacin-0.68; Trimethoprim/sulfamethoxazole-0.64 (Lin et al., 2024a)
	Klebsiella pneumoniae	Carbapenems-0.96 (Xu, 2024); 93%* (Huang et al., 2020); ceftazidime-avibactam-0.84 (Lin et al., 2024b)
Decision Tree	Escherichia coli	Carbapenems-0.95 (Xu et al., 2025)
	Klebsiella pneumoniae	Carbapenems-0.78 (Xu et al., 2025)
Random Forest	Staphylococcus aureus	Levofloxacin-0.95; Trimethoprim/sulfamethoxazole-0.95 (Lin et al., 2024a)
	Escherichia coli	carbapenems-1.00 (Xu et al., 2025)
	Klebsiella pneumoniae	carbapenems-0.95 (Xu et al., 2025); 94%* (Huang et al., 2020); ceftazidime-avibactam-0.95 (Lin et al., 2024b); Piperacillin/Tazobactam-0.96; Ceftazidime-0.90; Ceftriaxone-0.83; Cefotetan-0.93; Aztreonam-0.92; Imipenem-0.96;Amikacin-0.99; Levofloxacin-0.79; Co-trimoxazole-0.95 (Xu and Gao, 2025)
Gradient Boosting Machine	Staphylococcus aureus	Levofloxacin-0.95; Trimethoprim/sulfamethoxazole-0.93 (Lin et al., 2024a)
	Klebsiella pneumoniae	ceftazidime-avibactam-0.95 (Lin et al., 2024b)
	Escherichia coli	carbapenems-0.99 (Xu et al., 2025)
	Klebsiella pneumoniae	carbapenems-0.93 (Xu et al., 2025)
XGBoost	Staphylococcus aureus	Levofloxacin-0.94; Trimethoprim/sulfamethoxazole-0.94 (Lin et al., 2024a)
	Escherichia coli	carbapenems-0.99 (Xu et al., 2025)
	Klebsiella pneumoniae	carbapenems-0.90 (Xu et al., 2025); 0.97 (Xu, 2024); ceftazidime-avibactam-0.95 (Lin et al., 2024b); Piperacillin/Tazobactam-0.96; Ceftazidime-0.91; Ceftriaxone-0.89; Cefotetan-0.92; Aztreonam-0.95; Imipenem-0.96; Amikacin-0.99; Levofloxacin-0.80; Co-trimoxazole-0.95 (Xu and Gao, 2025)
Extremely Randomized Trees	Escherichia coli	carbapenems-1.00 (Xu et al., 2025)
	Klebsiella pneumoniae	carbapenems-0.95 (Xu et al., 2025)
Support Vector Machine	Klebsiella pneumoniae	carbapenems-0.97 (Xu, 2024); 87%* (Huang et al., 2020)
	Staphylococcus epidermidis	Rifampicin-0.92; Gentamicin-0.93 (Ren et al., 2024)
Multilayer Perceptron	Staphylococcus aureus	clindamycin-0.81; tetracycline-0.87; trimethoprim-sulfamethoxazole-0.72 (Wang et al., 2024)
AdaBoost	Staphylococcus aureus	Levofloxacin-0.89; Trimethoprim/sulfamethoxazole-0.86 (Lin et al., 2024a)
	Klebsiella pneumoniae	ceftazidime-avibactam-0.87 (Lin et al., 2024b); Piperacillin/Tazobactam-0.95; Ceftazidime-0.85; Ceftriaxone-0.87; Cefotetan-0.89; Aztreonam-0.92;Imipenem-0.96; Amikacin-0.98; Levofloxacin-0.81;Co-trimoxazole-0.93 (Xu and Gao, 2025)
Light Gradient Boosting Machine	Staphylococcus aureus	Levofloxacin-0.96; Trimethoprim/sulfamethoxazole-0.94 (Lin et al., 2024a)
	Klebsiella pneumoniae	ceftazidime-avibactam-0.95 (Lin et al., 2024b)
	Staphylococcus epidermidis	Ciprofloxacin-0.96; Rifampicin-0.92 (Ren et al., 2024)
Linear Discriminant Analysis	Staphylococcus aureus	Levofloxacin-0.67; Trimethoprim/sulfamethoxazole- 0.64 (Lin et al., 2024a)
	Klebsiella pneumoniae	ceftazidime-avibactam-0.80 (Lin et al., 2024b)
Naïve Bayes	Klebsiella pneumoniae	86%* (Huang et al., 2020)
Nearest Neighbors	Klebsiella pneumoniae	91%* (Huang et al., 2020)

“*” indicates the Accuracy metric, and unmarked entries indicate the AUROC metric.

### 3.4 Hyperparameter optimization

Hyperparameters are parameters that should be specified prior to model training. The optimization objective is to have the optimal combination of hyperparameters identified to maximize model performance on the validation set (Greener et al., 2022). Typically, after the hyperparameter grid is constructed, cross-validation is conducted to assess the performance of each hyperparameter configuration. The optimal hyperparameter combination is selected based on the cross-validation performance. Subsequently, the model is retrained on the entire training dataset using the selected optimal hyperparameter combination (Bzdok et al., 2018; Pfob et al., 2022).

### 3.5 Model training

The annotated mass spectrometry features are fitted to the “drug-resistant/susceptible” labels in the process of model training. The underlying patterns and regularities in the data are learned thereby, and outcomes for unseen data are enabled to be accurately predicted by the model. In the study by Nguyen HA et al., five base models-Logistic Regression, Random Forest, Support Vector Machine, LightGBM, and Multilayer Perceptron-were applied with different feature extraction methods (e.g., dynamic binning and latent embedding) to develop models (Nguyen et al., 2024).

### 3.6 Model validation and evaluation

The effectiveness and robustness of ML models are what the clinical applicability is substantially contingent upon. Consequently, a pivotal role is assumed by model validation and evaluation. During the model training phase, model validation is predominantly executed, and following model deployment, it can also serve as a continuous monitoring and validation mechanism. The primary objectives of model validation include the optimization of model hyperparameters being facilitated, overfitting or underfitting being averted, the model’s generalization capability being assessed, and robust performance on unseen data being ensured. Model validation is typically implemented using the validation set approach or cross-validation techniques. Validation accuracy, validation loss, learning curves, and cross-validation metrics are commonly employed as validation metrics.

The model’s overall performance is provided with a comprehensive and systematic assessment after the completion of model training through model evaluation, and its suitability for practical application is thereby determined. The test set approach, Leave-One-Out Cross-Validation (LOOCV), and cross-validation are commonly used as model evaluation methods. In addition to these methods, a variety of assessment metrics are involved in model evaluation, including sensitivity, specificity, accuracy, positive predictive value, negative predictive value, AUROC, AUPRC, and F1 score. Among these metrics, interpretable summaries of the model’s overall performance are offered by AUROC, AUPRC, and F1 score. Although still somewhat affected by class imbalance, these metrics’ ability to recognize minority classes is more accurately evaluated than that of traditional metrics, and thus the model’s performance on imbalanced data is better reflected by them. In cases of class imbalance, it is recommended to use a combination of multiple metrics to comprehensively evaluate model performance. Based on these evaluation metrics, continuous optimization of the model can be achieved, and model performance can be progressively enhanced through iterative refinement.

The aforementioned cross-validation can be involved in various stages within the process (Bradshaw et al., 2023). In the context of feature selection, the influence of different feature subsets on model performance can be evaluated by cross-validation, thereby allowing the most informative features to be selected. The comparison of various algorithms on the same dataset can also be facilitated by cross-validation, which allows for the identification of the algorithm best suited for a specific problem and dataset. Furthermore, model performance across different hyperparameter settings can be assessed by cross-validation, and the optimal hyperparameter combination can be identified thereby. During model evaluation, a more stable and reliable performance estimate can be provided by cross-validation through assessing the model on multiple training/testing splits (Bradshaw et al., 2023). K-fold cross-validation, LOOCV, and nested cross-validation are encompassed by common cross-validation techniques.

### 3.7 Model interpretation

The decisions and output results of a model can be clearly understood and explained, which is determined by the model’s interpretability (Allgaier et al., 2023). SHAP are derived from the concept of Shapley Value in game theory. The SHAP value for each feature can be calculated, and the contribution of each feature to the model’s prediction can be quantified, with both local and global interpretations being provided. A variety of visualization tools, such as Bar Plots and Bee Swarm Plots, are offered by SHAP to display the SHAP values of features, and the transparency and interpretability of ML models are thereby enhanced. The UniProt database is commonly used to identify proteins that are directly or indirectly related to AMR mechanisms. If certain peaks are not matched to known proteins, this suggests that unexplored MALDI-TOF MS data is indicated (**Table 2**).

**Table 2:** Resistance-associated peaks in select literature.

Microorganisms	Antimicrobial agents	Resistance (R)/susceptibility (S) prediction relevance	Peak (Da)
Staphylococcus aureus	Methicillin	R	(6,593.2) (Yu et al., 2022) (1975, 2134, 2592, 3890, 2204, 2410, 2874, 4607, 6594, 9216, 5053, 5541, 5579) (Kim et al., 2019) (3891, 4074, 1695, 2451, 2978) (Wang et al., 2018a) (2412) (Alksne et al., 2020)
		S	(6,550) (Yu et al., 2022) (2194, 2232, 2301, 2339, 2631, 2668, 3034, 5509) (Kim et al., 2019)
	Oxacillin	R	(3890, 5670, 2450, 3120, 4640, 9650) (López-Cortés et al., 2025) (6,593, 2,414, 2,432, 2,456) (Chung et al., 2021)
	benzylpenicillin	R	(6422.37, 4305.59) (Esener et al., 2021)
		S	(4807.21, 6891.17, 9621.26) (Esener et al., 2021)
	clindamycin	S	(2,414, 2,432, 2,456, 7,595) (Chung et al., 2021)
	erythromycin	S	(6,593, 2,413, 2,432, 2,456) (Chung et al., 2021)
Staphylococcus epidermidis	Ciprofloxacin	R	(7,748, 6,812, 7,780, 8,077, 7,460) (Ren et al., 2024)
	Gentamicin	R	(7,753, 9,233, 2,979, 10,572, 3,877) (Ren et al., 2024)
	Rifampicin	R	(8,077, 8,107, 5,726, 16,210, 7,766) (Ren et al., 2024)
Enterococcus Faecalis	Vancomycin	R	(3,302, 6,603) (Wang et al., 2022) (3,172, 3,302, 3,645, 6,342, 6,356, 6,603, 6,690) (Wang et al., 2021)
		S	(3,165, 3,681, 7,360) (Wang et al., 2021)
Escherichia coli	Ciprofloxacin	R	(5890) (López-Cortés et al., 2025) (6809) (Chung et al., 2023b)
		S	(5230, 3850, 9225) (López-Cortés et al., 2025) (7650, 10534, 11783) (Chung et al., 2023b)
	Carbapenems	R	(7,870-7,879) (Xu et al., 2025)
Klebsiella pneumoniae	ceftazidime-avibactam	R	(2024, 3095, 4518, 5416, 5497, 6300, 7124, 9593) (Lin et al., 2024b)
	Ciprofloxacin	R	(6590, 5065, 6550, 4515) (López-Cortés et al., 2025)
	Carbapenems	R	(4497.59, 4547.15, 5040.46, 8876.78, 8993.44, 9139.06) (Ye et al., 2025) (4521) (Huang et al., 2022) (4,920-4,929) (Xu et al., 2025) (2480, 4967, 12,362, 12,506, 12,855, 14,790, 15,730, 16,176, 16,218, 16,758, 16,919, 17,091, 18,142, 18,998, 19,095) (Zhang et al., 2023) (4519) (Xu, 2024) (2764, 3150, 4605, 5822, 6152) (Jian et al., 2024)
	Colistin	R	(4154, 4341, 4571, 5252, 6596, 7158, 8993, 9252) (Jian et al., 2024)
Campylobacter	Ciprofloxacin	R	(6436.22, 2766.98, 2241.84, 3257.41, 7083.30) (Feucherolles et al., 2021)
	Tetracycline	R	(4365.25, 2766.98, 7083.30, 6436.22, 2713.95) (Feucherolles et al., 2021)
Mycobacterium abscessus	amikacin	S	4,062.03 (Thai et al., 2025)
	linezolid	R	7,518.33 (Thai et al., 2025)
	clarithromycin	R	8,359.34 (Thai et al., 2025)
	cefoxitin	R	2,493.06 (Thai et al., 2025)
Stenotrophomonas maltophilia	trimethoprim/sulfamethoxazole	R	(6,528, 7,376, 7,172, 5,725, 9,589, 8,488, 5,115, 4,266, 3,683, 2,427) (Lin et al., 2024a)
	levofloxacin	R	(5,269, 6,100, 6,528, 7,172, 5,725, 7,376, 8,488, 5,115, 4,266, 2,427) (Lin et al., 2024a)
Aspergillus fumigatus	Azoles	R	(7087.4, 14169.6, 6867.2, 6582.2, 3011.7, 3114, 3606.4, 2261.1, 9627.7, 5023.7, 7732.7, 4578.1, 2999.9, 4586.2) (Zvezdanova et al., 2022)
Burkholderia pseudomallei	ceftazidime, meropenem, imipenem, co-amoxiclav, co-trimoxazole	R	(2250.52, 2272.25, 2362.63, 3151.96, 3977.36, 4072.81, 4605.19, 7536.54, 9732.58, 12468.3) (Nithimongkolchai et al., 2025)

The dissemination pathways of resistance genes can be tracked by MALDI-TOF MS through the detection of proteins that are encoded by these genes. Bacterial resistance can be caused by spontaneous mutations within their own genomes and by resistance genes that are acquired from other bacteria via horizontal gene transfer. For instance, the PSM-mec peptide, which is encoded by the *mecA* gene-a key determinant of oxacillin resistance in *staphylococci*, can be detected by MALDI-TOF MS as utilized by Weis et al (Weis et al., 2022). The PSM-mec peptides are detected in diverse bacterial species, which suggests that the *mecA* gene may be harbored by these bacteria. The *mecA* gene could have been acquired through horizontal gene transfer mechanisms, such as plasmid dissemination. Indirect inferences regarding the dissemination of antibiotic resistance genes can be made based on this observation. While a ciprofloxacin resistance model was being investigated, a protein associated with the characteristic bin 2,025 was identified by researchers. Although this protein was not annotated as a protein of *Staphylococcus epidermidis* in the UniProt database, it was identified as a member of the pathogenicity island family in *Staphylococcus aureus* at a molecular weight of 8,077 Da (Ren et al., 2024). The capacity of MALDI-TOF MS to identify pertinent biomarkers is underscored by this.

These biomarkers are frequently associated with known resistance mechanisms, either directly or indirectly. However, considering the complex nature of protein functions and interactions, the proteins that confer resistance to microorganisms may not necessarily be the identified biomarkers. Rather, they may be encoded by the same gene or gene cluster that encodes the proteins responsible for resistance (Feucherolles et al., 2021). The accurate identification of resistance-associated proteins can be complicated by overlapping mass spectral peaks, which can be caused by the highly similar molecular weights of distinct proteins in MALDI-TOF MS data (Nguyen et al., 2024). Nonetheless, the precise identification of resistance-associated proteins or genes can be achieved by employing molecular docking simulations (Yu et al., 2022)or by integrating genomic sequencing data (Jiang et al., 2019). Additionally, molecular weights that exceed the conventional detection range of MALDI-TOF MS (approximately 2,000 - 20,000 Da) may be possessed by certain resistance-determining proteins. In such instances, surrogate biomarkers may be detected to serve as an indirect means for resistance to be inferred. For example, in *Staphylococcus aureus*, the molecular weight of the mecA protein, which confers resistance to methicillin, is beyond the conventional detection range. However, its smaller product, PSM-mec (with a molecular weight of 2,415 Da), remains within the detectable range and can be utilized to identify strains harboring the *mecA* gene (Rhoads et al., 2016). To ensure the veracity and robustness of predictive interpretations, the biological functions of identified features should be prudently elucidated.

### 3.8 Model application and update

Significant challenges in infrastructure, finance, and training may be posed by the integration of MALDI-TOF MS and ML into clinical practice. To justify the investment, it is essential to conduct rigorous cost - effectiveness studies. Additionally, the current insufficiency in the availability of open - source data and code can be addressed by establishing large - scale, publicly available datasets and analytical codes. Model validation and optimization by other researchers would be facilitated by this approach, thereby enhancing the reproducibility of studies (Weis et al., 2020). MALDI-TOF MS data of AMR are highly geographically specific. Pretrained models, which are trained on other datasets, cannot be directly applied and need to be adjusted according to local data. Moreover, the resolution, peak intensity, and noise of mass spectra may be varied by different instruments. Pretrained models need to be fine - tuned for a specific setting to be effective (López-Cortés et al., 2025). Therefore, the standardization of data and processes should be focused on by future research to enable transfer learning across different regions and instruments. The development of models that are adaptable to diverse clinical environments and the improvement of the accuracy of AMR prediction will be facilitated by this. Given that AMR is dynamically evolving, it is essential that continuous data monitoring and dynamic model updating be carried out to ensure the validity and clinical value of these models. A multimodal feature repository can be established by integrating proteomic fingerprints with patient-derived genomic profiles and clinical metadata, and its online, continuously updated framework can be advanced in parallel with privacy-compliant information sharing. The feasibility and effectiveness of using MALDI-TOF MS integrated with ML for AMR prediction can be further enhanced by future research through expanding the range of species studied, extending MALDI-TOF MS datasets, integrating multimodal data, and consolidating clinical information (Peiffer-Smadja et al., 2020).

## 4 Conclusion

More rapid AMR information than that provided by conventional methods is offered by the integration of MALDI-TOF MS with ML, which facilitates automated data processing and presents a promising prospect for rapid and accurate AMR prediction. ML models with high training efficiency, interpretable results, and strong generalization ability need to be constructed as a key step to ensure rapid and accurate AMR prediction using this technology. The acquisition of raw data, preprocessing of raw data, algorithm selection, hyperparameter optimization, model training, model validation and evaluation, model interpretation, and model application and updating are all included in the multiple steps of the modeling process. These steps can be dynamically adjusted to be carried out in parallel or alternately to achieve the best model performance and ensure the flexibility and adaptability of the process. Despite challenges such as imbalanced data categories, dynamic changes in AMR, and insufficient model generalization and interpretability, great potential is held by future optimization measures. By standardizing spectral acquisition and preprocessing procedures, collecting high-quality and large-scale MALDI-TOF MS datasets, open-sourcing datasets and model codes, training highly specialized and generalizable models, exploring multimodal data fusion, and implementing transfer learning and application across different clinical centers, this technology is expected to become an important tool for AMR detection and antimicrobial stewardship.
